# Work Aspects Related to and Protective of Nurse Burnout During the Pandemic: A Cross‐Sectional Study

**DOI:** 10.1155/jonm/1851095

**Published:** 2026-02-13

**Authors:** Carolyn M. Porta, Mark Linzer, Roger Brown, Jordyn M. Deubel, Erin E. Sullivan

**Affiliations:** ^1^ School of Nursing, University of Minnesota, Minneapolis, Minnesota, USA, umn.edu; ^2^ Department of Medicine, Hennepin Healthcare and University of Minnesota, Minneapolis, Minnesota, USA; ^3^ School of Nursing, University of Wisconsin, Madison, Wisconsin, USA, wisc.edu; ^4^ Sawyer Business School, Suffolk University, Boston, Massachusetts, USA, suffolk.edu; ^5^ Center for Primary Care, Harvard Medical School, Boston, Massachusetts, USA, harvard.edu

## Abstract

**Aim:**

To determine work conditions and reactions related to nurse burnout while exploring characteristics of favorable work environments among nurses without burnout.

**Design/Methods:**

The Coping with Covid survey, administered between April 14, 2020, and March 2021, was fashioned after the Mini Z, with 15 questions related to work conditions (fear/safety, work overload, anxiety/depression from work, feeling valued, and sense of purpose) and healthcare worker (HCW) reactions, including burnout, stress, and intention to leave (ITL) the job. Work conditions and reactions such as burnout were measured on 5‐point scales and then dichotomized as present/high or absent/low. Multilevel regressions assessed burnout‐related work conditions, adjusting for gender, race, and years in practice. Thematic analysis of comments from nurses without burnout identified protective factors.

**Results:**

Of 58,408 HCW respondents at over 200 organizations (median response rate 34%), 11,040 were nurses, with 10,873 at 43 organizations with over 10 nurses. More than half the nurses (56%) reported burnout, 42% intended to leave, and only 40% felt valued. Nurse burnout was significantly related to workload (adjusted odds ratio (aOR) 3.71 (95% CIs 3.26, 4.22), *p* < 0.001); anxiety/depression symptoms from work (aOR 2.96 (2.59, 3.39), *p* < 0.001), and fear of exposure to SARS‐CoV‐2 (aOR 1.38 (1.20, 1.59), *p* < 0.001). Those feeling valued had less than half the odds of burning out (aOR 0.40 (0.35, 0.46), *p* < 0.001). These variables explained 42 percent of burnout variance. Nurses without burnout identified additional positive influences, including teamwork, leader support, and timely communication.

**Conclusions:**

Nurse burnout may be prevented by addressing workload, mental health symptoms, and relationships with team members and leaders. Feeling valued is a strong mitigator of burnout. These strategies may be useful to nurse leaders in building sustainable workplaces.

**Implications:**

Almost half of nurse burnout may be addressed by a brief list of remediable variables.

## 1. Introduction

Five years before the onset of the COVID‐19 pandemic, a systematic review summarized 25 years of research on burnout among emergency department nurses [1]. One in four nurses experienced burnout, which was predicted and prevented by work environment factors including teamwork and nurse‐leader relationships. Burnout among nurses is not novel; what is new is increased visibility of the problem and our collective determination to eliminate burnout and system‐level factors that cause it. Notably, today’s nursing workforce may be increasingly unwilling to remain in work environments that contribute to burnout [2], where nurses feel under‐resourced, unsafe, and not supported.

The SARS‐CoV‐2 pandemic tested the capacity of the global healthcare workforce, and their efforts, challenges, and exhaustion were visible on mainstream and social media; these impacts were well documented within and outside the United States [3, 4]. With nurses comprising the largest proportion of the workforce, their collective health and threats to health have garnered public attention since the early days of the pandemic and continue to do so today. This attention to nurse burnout positions us to explore moonshots—ambitious projects—aimed at eliminating burnout and sustaining the health of our workforce, including but not limited to nurses.

Global attention to the health of the nursing workforce [5, 6], and subsequently, to challenges to health including burnout, is also driven by projected workforce needs (12 million more nurses needed globally by 2035). In the United States (US), sophisticated analyses project a registered nurse (RN) shortage by 2030 of 63,720 full‐time equivalents (FTEs) that transitions to a modest excess by 2035 (16,180 FTEs) [7]. Given known links between burnout and intention to leave (ITL) the job, a focus on staff shortages appears to be a necessary accompaniment to studying burnout. Alzailai and colleagues address staff shortages in their work with ICU nurses in Saudi Arabia [8], identifying important areas such as family context, work context, lack of support, and discrimination. State‐level shortages and excesses vary widely between now and 2035, as do within‐state estimations for micro‐ and metropolitan geographic settings. Factors related to projected shortages have been recently published [9], although U.S. nursing workforce cyclical shortages have been documented since the 1930s [10]. Nonetheless, threats to nurses’ health, exacerbated by the pandemic and its aftermath (long Covid, new viral threats, caregiver burdens, post‐traumatic stress, and economic strains), must be understood and intervened upon.

In the COVID‐19 Impact Assessment Survey by the American Nurse Foundation and the American Nurse Association, 60% of acute care nurses reported burnout [11, 12], 75% reported feeling exhausted, and half (52%) considered leaving their position [12]. Khatatbeh and colleagues studied relationships between burnout and overall health and found work shifts (rotating night and day) were important covariates in the burnout‐health relationship [13]; they also found a relationship between pediatric nurses’ quality of life and ITL (negative modest correlation, *r* = −0.227, *p* < 0.01) [14]. Liao and colleagues studied burnout, occupational commitment, and social support among Chinese pediatric nurses and found commitment and social support moderated the relationship between stress and burnout [15]. An Australian team found 61% of 762 nurses were affected by bullying, which had a moderate correlation with burnout (0.38, *p* < 0.001) [16]. An early pandemic systematic review [17] of 18,000 nurses found several occupational factors (including workload) and several other psychosocial contributors to nurse burnout. Another review in 2021 [18] assessed risks for PTSD in nurses and found a relationship to anxiety and depressive symptoms. Aiken and colleagues analyzed 15,738 nurses in 60 U.S. Magnet (high quality) hospitals early in the pandemic and found 47% reported burnout (twice the pre‐pandemic level), which was associated with turnover of both nurses and physicians [19]. Many nurses (26%) rated hospitals unfavorably for patient safety, one‐third described poor work environments, and 87% described a need to improve nurse staffing.

With calls for randomized intervention trials to reduce nurse burnout by the U.S. National Institute of Health, it is imperative we identify malleable contributors. We thus performed a secondary analysis of data from the Coping with Covid study [20] to identify (1) prevalence of nurse burnout and ITL; (2) work conditions associated with burnout; and (3) characteristics of favorable work environments in nurses not experiencing burnout. Special attention was paid to known and hypothesized variables of high relevance to nursing, including workload [17], safety [19], mental health support [18], and the experience of feeling valued [20]. Each of these has been related to nurses’ experiences during and, in most cases, outside the pandemic. A mixed methods approach, with quantitative and qualitative aspects, was chosen to allow amplification and contextualization of quantitative findings on burnout predictors by bringing in the open‐ended comments and perspectives of nurses not suffering from burnout.

## 2. Methods

### 2.1. Study Design

The Coping with Covid study design [21, 22] is theoretically grounded in the Demand‐Control Model of Job Stress [21–23], which facilitates inquiry into factors driving stress and burnout. At the outset of the pandemic, we created a short “Coping with Covid” survey modeled after the Mini Z [24] (see below), which was rolled out quickly in a convenience sample and cross‐sectional manner to healthcare organizations across the United States affiliated with the American Medical Association (AMA) and to institutions that heard through word of mouth. Information was on a public website, and organizations required more than 100 physicians to participate. The number of questions varied as some were added through the pandemic (e.g., burnout [April 2020], work intentions [summer 2020], and feeling valued [fall 2020]). There were 20 questions, including demographics, allowing for a quick response.

### 2.2. Measurement

Burnout was measured with a single item, validated against the Maslach Burnout Inventory Emotional Exhaustion subscale (Pearson correlation 0.64, *p* < 0.001 [25]), and scored from no burnout (responses 1 and 2 on the 5‐choice scale) to burnout (top 3 choices, all mentioning the word “burnout”). Work environment aspects were measured on 4‐point scales from low to high (1. *Not at all*, 2. *Somewhat*, 3. *Moderately*, and 4. *To a great extent*), and included fear of exposure (safety), anxiety/depression due to COVID care (mental health symptoms), work overload, sense of mission and purpose, and feeling valued by the organization. Nurse reactions (burnout, intention to reduce hours, and ITL) were measured on 5‐point scales. Four‐choice items were modeled after single items within the validated Mini Z measure [24] and have shown significant correlations with burnout in several Coping with Covid analyses for varied healthcare worker (HCW) roles [20]. As has been the convention for all Coping with Covid analyses [20], and in early related work in the Physician Worklife Study [26], the upper two responses (*moderately* and *to a great extent*) in four‐choice items were considered *high* or *present.* A stress summary score of four of these items had a Cronbach’s alpha within the physician sample of 0.72 and was also reasonable for the full nurse sample in the current study at 0.75. Five‐choice items were also adapted from the Physician Worklife Study [26] and determined burnout, intention to reduce hours, and ITL, which correlates with burnout [19, 20]. In 5‐choice items, the top 3 choices were considered *high* or *present*. Relevant questions from the survey are found in Supporting Table 1.

### 2.3. Population

Coping with Covid addressed work conditions and outcomes for all HCW roles, ranging from nurses and doctors to physical therapists and food service. Early findings showed variability in work conditions between groups, with nurses reporting some of the higher rates of burnout [20].

### 2.4. Sampling

Any hospital with more than 100 employed physician providers was eligible. Different hospitals enrolled different HCW roles with a wide variability in the number of nurses or other HCWs enrolled at each site. Hospitals with smaller numbers of nurses (*n* < 10) were excluded from the regression analyses based on the multilevel modeling requiring nested nurse numbers of 10 or more nurses per organization to adequately estimate model variability.

### 2.5. Data Collection and Timing of the Study

Surveys were fielded online by individual organizations between April 2020 and March 2021. Organizations administered their own surveys and determined the timing of reminders. Surveys were administered at no cost to the organizations, with no incentives provided for participation. Responses went to the affiliated data lab, Forward Health Group, in Madison, WI, United States.

A *mixed methods* (quantitative and qualitative) approach was chosen to amplify findings from the quantitative assessment; multilevel regression modeling was thus used first to determine a set of key variables related to burnout, followed by a thematic analysis of open‐ended comments from nurses without burnout to determine additional protective factors that may have been important to them.

*Quantitative analyses* included descriptive statistics and multilevel (two‐level analyses with nurses nested within organizations) logistic regression models; regressions adjusted for gender, race, and years in practice to determine factors associated with burnout and a pseudo r‐square (McKelvey & Zavoina’s r‐square) for the percent of burnout variance explained by these variables. Variables added later (ITL and feeling valued) had excellent response rates (99%). Virtually all variables had very low missingness (< 1%), and imputation was not attempted. A covariate was created for Covid hospital exposure rates, expressed as daily new cases per 100,000 people (7‐day moving average) per organization location by county from July 1 to December 31, 2020; this variable was then added to the regression models as a covariate. Missingness was higher in this variable (32%), as it came from incomplete national databases. Because this variable served as an adjusting covariate in a large complete‐case sample, we elected to conduct a complete‐case analysis to preserve interpretability and avoid model‐driven imputation, thus prioritizing internal validity. Hospitals with less than 10 nurses were not included to allow for stable and more precise multilevel modeling. The final sample of nurses at hospitals with 10 or more nurses was *n* = 10,873; data from these nurses were available for use in the regressions. Comparisons were made between burnout and ITL rates between nurses, physicians, and other clinical staff. A *p* value of < 0.05 was considered statistically significant in all comparisons.

### 2.6. Qualitative Analyses

We also analyzed nurses’ answers to the open‐ended question, “What else would you like to tell us about your experience during the COVID‐19 crisis?” We focused on comments from respondents in the lowest tercile of burnout scores. This group included nurses who reported neither burnout nor stress (choice 1 on the 5‐point scale) or who reported stress without burnout (choice 2). Data from these nurses yielded 1315 codable comments after excluding 248 incomplete or irrelevant comments, such as “nothing” or “N/A.” A single coder (ES) organized comments in Excel and performed a thematic analysis of the comments from nurses without burnout, using inductive coding to identify emerging issues related to nurses’ perceptions of work factors associated with well‐being; these were used to create an initial codebook, which was applied to the 1315 comments by the same coder (ES) [27]. A second coder (JD) reviewed 20% of the comments, confirming the findings (intercoder reliability, Cohen’s kappa = 0.9), and the two coders worked collaboratively to classify codes into themes [28]. This coding process overlapped with the standard components described by Braun and Clarke, including generating codes, organizing them into themes, and then naming the themes [29]. Preliminary findings and themes were discussed with two co‐authors (ML, CMP) and further categorized in an ecologically driven framework consisting of macro‐, micro‐, and individual levels [30].

### 2.7. Ethical Review

Data used in this analysis were collected as a quality improvement program originally determined to be low risk (no intervention, survey‐based, de‐identified data) and were felt not to be Human Subjects Research by Hennepin Healthcare’s Institutional Review Board (exemption provided on March 23, 2020). Completing the survey was considered assent and agreement to participate.

STROBE guidelines for an observational study were generally followed for the Coping with Covid study. A checklist is included in the Supporting information.

## 3. Results

Of 58,408 total survey respondents from over 200 U.S. institutions, 11,040 (18.9%) were nurses. After excluding organizations with 10 nurses or less, there were 10,873 nurses for analysis at 43 organizations (85% female, 67% White, and 37% in practice for over 20 years; see Table 1). Organizations were found in varied locations, with 42% in the Northeast, 26% in the Midwest, 16% in the South, and 14% in the Western United States; one system (2%) was outside the United States. Over half (56%) of the nurses reported burnout (significantly higher than rates in physicians or other clinical staff; see Supporting Figures 1A and 1B). Work conditions were often unfavorable, including moderate or higher ratings of fear of exposure to SARS‐CoV‐2 (69%), work overload (47%), and work‐related anxiety/depression (44%). Childcare stress was noted by 1 in 5 nurses (20%), while a sense of meaning and purpose (*moderate* or *to a great extent*) was reported in 52%. A known burnout mitigator, feeling valued [31], was present (*moderate* or *to a great extent*) in only 40% of nurses. Thirty‐six percent of nurses intended to reduce work hours. Forty‐two percent intended to leave, which was significantly more than the rate among 13,780 physicians and 5415 other clinical staff (e.g., medical assistants and social workers; see Supporting Table 2 and Figures 1A and 1B).

**Table 1:** Demographics and work conditions for the nursing burnout study, 2020–21.

	Frequency count	Percent (%)	Percent less missing (%)
Sex			
Prefer not to answer	911	8.4	8.4
Male	724	6.7	6.7
Female	9214	84.7	84.7
Nonbinary/third gender	25	0.2	0.2
Race and ethnicity			
Missing	898	8.3	
Prefer not to answer	1337	12.3	13.4
White	6729	61.9	67.5
Hispanic/Latino	323	3.0	3.2
Black/African American	765	7.0	7.7
Native American or American Indian	18	0.2	0.2
Asian/Pacific Islander	684	6.3	6.9
Other (please specify)	120	1.1	1.2
Years in practice			
Missing	201	1.9	
1–5 years	2231	20.5	20.9
6–10 years	1888	17.4	17.7
11–15 years	1506	13.9	14.1
16–20 years	1080	9.9	10.1
More than 20 years	3968	36.5	37.2
Burnout			
Missing	898	8.3	
Absent	4373	40.2	43.8
Present	5603	51.5	56.2
Intention to leave practice^a^			
Missing	8670	79.7	
Low	1288	11.8	58.4
High^b^	916	8.4	41.6
Intention to reduce work hours^a^			
Missing	8728	80.3	
Low	1379	12.7	64.3
High^b^	767	7.1	35.7
Stress			
Missing	1	0.01	
Low	7212	66.3	66.3
High	3661	33.7	33.7
Fear of exposure			
Low	3385	31.1	31.1
High	7489	68.9	68.9
Anxiety/depression from work			
Missing	4	0.1	
Low	6082	55.9	56.0
High	4788	44.0	44.0
Workload			
Missing	4	0.1	
Low	5766	53.0	53.0
High	5104	46.9	47.0
Childcare stress			
Missing	12	0.1	
Low	8729	80.3	80.4
High	2133	19.6	19.6
Feeling valued			
Missing	4	0.1	
Low	6516	59.9	59.9
High	4354	40.0	40.1
Meaning and purpose			
Low	5266	48.4	48.4
High	5608	51.6	51.6

^a^Large number of missing values due to these questions not being asked of all organizations.

^b^High = moderately, likely, or definitely (for intent to leave and intent to reduce hours) on a 5‐item scale. High for other categories means the top two choices on a 4‐point scale.

Regression analyses in 6597 nurses in 29 organizations with complete data (Table 2) identified stress, personal safety, work overload, and anxiety/depressive symptoms as strong burnout correlates (adjusted odds ratios (aORs) ranging from 1.38 to 3.71, *p*’s < 0.001); nurses feeling valued by their organizations had less than half the odds of burning out (aOR 0.40, 95% CIs 0.35, 0.46, *p* < 0.001). The model’s pseudo *r*‐square was 42%, suggesting close to half of burnout variance was explained by these variables.

**Table 2:** Work–life variables associated with burnout in the Coping with Covid study, April 2020–March 2021.

	**Predictors of burnout in nurses**		
	**Adj OR**	**p > |*Z*|**	**95% CI**
Stress			
High^∗^	2.38	< 0.001	2.06, 2.76
Fear of exposure			
High	1.38	< 0.001	1.20, 1.59
Anxiety/Depression			
High	2.96	< 0.001	2.59, 3.39
Workload			
High	3.71	< 0.001	3.26, 4.22
Childcare stress			
High	0.98	0.84	0.84, 1.15
Meaning and purpose			
High	0.93	0.29	0.801, 1.06
Feeling valued			
High	0.40	< 0.001	0.35, 0.46
Ethnicity			
White	0.87	0.24	0.70, 1.09
Hispanic/Latino	0.77	0.15	0.53, 1.10
Black/African American	0.56	< 0.001	0.43, 0.75
Native American	0.43	0.33	0.08, 2.35
Asian/Pacific Islander	0.69	0.01	0.52, 0.92
Other	0.70	0.20	0.41, 1.20
Sex			
Male	0.85	0.35	0.60, 1.19
Female	0.90	0.43	0.68, 1.18
Nonbinary/third gender	1.23	0.75	0 0.35, 4.37
Years in practice			
6–10 years	1.22	0.06	0.99, 1.50
11–15 years	1.12	0.31	0.90, 1.38
16–20 years	0.89	0.31	0.70, 1.12
More than 20 years	0.80	0.01	0.67, 0.94
Covid rate	1.01	0.50	0.989, 1.03
Constant	0.50	0.01	0.32, 0.79
Organization Var	0.04		0.01, 0.11
McKelvey & Zavoina’s R2	0.42		

*Note:* Number of nurses with complete data at hospitals with 10 or more nurses, 6597 with 29 organizations represented in the regressions. Multilevel regressions were adjusted for gender, race, years in practice, and Covid hospital rates to determine factors associated with burnout. Much of the missing data were in Covid hospital rates.

^∗^“High” = response of moderate or to a great extent.

Comments from 1315 nurses without burnout (see Consort Diagram, Supporting Figure 2) generally aligned with quantitative results, although these nurses were less likely to mention unfavorable work conditions, such as fear of exposure, work overload, or need for mental health support. New insights related to several favorable work conditions that are mapped to our ecological framework, including individual, relational, and system‐level factors, are below (see Table 3 for codes and major themes).

**Table 3:** Mapping of codes and themes to ecological framework categories.

Code	Theme	Ecological framework level
Increased sense of purpose	Sense of purpose	Individual
Values connection		

Importance of patient care	Passion for patient care	Individual
Caring for COVID patients		

Burnout resolving	Professional growth	Individual
Learning new skills		

Missed the COVID wave	Not assigned to COVID care	Individual
No COVID patients		

Need more communication	Transparent communication	Relational
Need transparency		
Poor communication causes stress (negative)		

Team member care and support	Teamwork	Relational
Team adapted		
Complaint about coworkers (negative)		

Supportive leaders, managers, or administrators	Leader support	Relational
Supported by additional help or resources		

Appreciated	Appreciation	Relational
Felt valued		
Not appreciated (negative)		

Need for PPE	PPE	System
Reuse of PPE		

Supportive organization	Organizational support	System
The organization handled the crisis well		
Lack of caring by the organization		

Anxiety about getting COVID	Organizational support (safety)	System
COVID exposure		
Got COVID		

Feel safe at work		

Need arrangements for pre‐existing conditions	Organizational support (resources)	System
Need time off		
Need hazard pay		
Need mental health support		

Family impact	Organizational support (work–life balance)	System
Concern about day care		

Isolation	Organizational support (work environment)	System
Clinic less stressful		
Workload		
Need more staff		
Need support for new nurses		

Uncertainty about guidelines	Rapid policy changes	System
New ways of doing things		
Complaint about masking policy		
Protocols make things less stressful		

Redeployment requiring new skills	Redeployment worries	System

Pay cuts, furloughs	Adverse financial impact	System
Worried about job security		

Abbreviation: PPE = personal protective equipment.

For nurses without burnout, *individual-level factors* explaining low burnout included sense of purpose, passion for patient care, and COVID as an opportunity to develop new skills (professional growth). One nurse explained, “I feel privileged that I am and was in a position to help people suffering from the pandemic. I love what I do, I love taking care of people. I don’t mind the increase in workload since my job is my passion…” Others described positive feelings related to professional development: “It was a positive experience for me. I learned so [many] skills during this time.” Some respondents reported not caring for COVID patients, not experiencing distress, or that burnout, if present, was short‐lived.

*Relational factors* included relationships with teams, managers, leaders, and administrators. The importance of the team coming together during the crisis and concerns for co‐workers were evident in positive comments similar to this exemplar statement, “There were definitely stressful days in the beginning, but we have a great team here and all work together to get through it.” Leadership support was highly valued, especially for leaders who demonstrated appreciation and valuing nurses during the crisis. Respondents praised transparent communication, preferring frequent (i.e., daily) updates related to workflow changes, and perceived this as decreasing their stress.

*System-related* factors were often communicated as sources of stress or frustration. Respondents noted organizational support was missing in their workplaces, including shortages of PPE, staffing, childcare, and hazard pay. Many felt “whiplash” in how fast guidelines changed, struggling to remember the latest recommendations. Nurses found furloughs financially burdensome, and several felt underutilized. One nurse explained, “I was more stressed when I was furloughed because I felt my RN experience was not being utilized.” Needs were expressed for workload adjustment and mental health support. Nurses found redeployment stressful, particularly if redeployed from outpatient to inpatient roles or deployed to work with new patient populations (i.e., pediatric nurses caring for adults). Positive comments credited organizations for caring: “I felt as if the organization took good care of us through the crisis.”

## 4. Discussion

In this secondary analysis of data from over 10,000 nurses collected during a challenging phase of the pandemic, we found a high number reported burnout and ITL. Related work conditions included workload, fear (e.g., personal safety), and anxiety and depressive symptoms due to work, confirming in nurses key factors contributing to burnout among other HCWs [32]. We offer unique findings that demonstrate a brief set of quantitative variables that explain almost half of nurse burnout. This parsimonious list of remediable work factors may inform nurse leaders of new mechanisms to improve work conditions, reduce burnout, and improve retention. Our quantitative data demonstrated that feeling valued by one’s organization, a known mitigator of burnout [31], was felt by less than half of our respondents, an unfortunately low finding given the very high levels of fear for safety, high workload, and mental health symptoms. Equally important was that those feeling valued had less than half the odds of burning out versus those not feeling valued; recently published work [33] highlights work factors, such as ensuring safety, empathic leadership, enhanced teamwork, and transparent communication, that may contribute to workers’ feeling valued. Comments from nurses without burnout added critical depth and nuance, both confirming and extending prior research. These nurses’ comments provided insight into components of favorable workplaces. Nurses without burnout described *individual-level* factors highlighting the protective role of developing meaning and purpose, preserving the passion for patient care, and cultivating professional growth and development. These well‐known contributors to a meaningful work life can be overlooked during a crisis; indeed, even in more routine practice, they are often ignored due to a focus on schedules, coverage, and benefits. Nurses reinforced that purpose, passion for care, and professional growth are key factors in a supportive work environment that engenders a desire to stay and flourish within an organization. Meanwhile, teamwork, leadership, and transparent communication emerged as powerful *relational factors*, underscoring the need for enabled and efficient teams, engaged and supported leaders, and timely, transparent information transfer from organizational leadership. These data reveal important opportunities to consider interventions that reduce negative exposures while cultivating positive individual, relational, and system‐level experiences. Our findings provide directions for future research and healthcare system strategies to support the nursing workforce and eventually contribute to eliminating nurse burnout.

In the relevant literature, a comprehensive review of 91 articles on burnout in nursing in 2020 [5] found several variables related to burnout that overlap with ones noted here, including workload, work control, and values alignment (from our quantitative analysis), and positive leadership and teamwork (from our qualitative analysis). Systematic reviews during the pandemic also identified psychosocial demands and confirmed workload as a deleterious factor [17, 18]. *Our findings move the field forward by identifying a short list of remediable factors amenable to further study and intervention. These factors include ensuring nurses’ safety, right-sizing workloads, a need for mental health support, and enabling leaders to communicate effectively, support a passion for patient care and promote a sense of being valued by one’s organization.*

Recent literature [14, 17, 18, 34] plus our findings offer insights into fruitful areas for change. The qualitative data in Table 3 highlight both challenges and opportunities in terms of personal safety, workload, and mental health symptoms, with a strong signal of the value brought by leadership, teamwork, communication, and organizational support. Work volume requires attention, with consideration given to customizing workloads to individual nurses’ capacity for assuming more work and adjusting staffing for patient complexity and illness acuity. Likewise, mental health sequelae, known to worsen because of vicarious (secondary) trauma [35], require breaks and debriefing as well as psychological support in real time following difficult workplace incidents [36]. In addition, safety remains a major factor for nursing in U.S. hospitals, and targeted initiatives are needed to identify high‐risk scenarios, provide alert systems (e.g., panic buttons), and adjust workflows to prevent violence.

Several other domains were also highlighted in Table 3. Developing venues where nurses can be more in touch with their own sense of purpose and their passion for patient care was strongly endorsed, reflecting a wish for relational over transactional workplaces; often lost in the focus on daily work schedules is a parallel need for professional growth and development. Finally, work–life balance is a well‐known contributor to thriving in the workplace, with a very strong correlation found between work–life interference and burnout in Boamah’s study of Canadian nurses [37]. Explicit attention to how successful we are in achieving these workplace modifications could pay large dividends in terms of satisfaction, lower burnout, and greater intention to stay with the organization.

*Future intervention studies* should test institution‐level strategies that result in healthy workplaces, including staff safety [38], right‐sized workloads (e.g., workload customization based on work capacity and patient acuity and staffing ratio assessments in varied settings), and protection of mental health via buddy, peer support, and Critical Incident Support programs. Strengthening protective factors, including high‐quality leadership [39] and system redesigns for teamwork, is critical to ensuring nurse well‐being. Additional research should examine which threats to nurses’ well‐being have persisted beyond the immediate postpandemic era. Burnout existed long before the pandemic, and without substantial system changes, will continue to do so.

This report has both *strengths and limitations*. Strengths include theory‐guided inquiry, use of many validated items, the focus on remediable factors, the very large national sample, and a reasonable response rate for a national survey. Limitations include a convenience sample using a brief survey with relatively few explanatory variables during a highly stressful (atypical) time, although the percentage of burnout variance explained was meaningful. There was little knowledge of institutional characteristics, and thus organizations involved cannot be described in detail. While many survey items were relatively new and incorporated at the onset of the pandemic, prior and current analyses have shown these items are linked to the validated burnout question.

## 5. Conclusions and Implications

There is an urgency to address nurse burnout. The parsimonious list of variables provided could potentially prevent burnout in nurses during times of societal stressors and strain. Workplace factors matter, such as workload and feeling valued. Systems that value worker health and employ trauma‐intelligent solutions will likely see benefits such as retention and longevity in the workforce. The benefits of addressing anxiety and depression and the effects of vicarious trauma in nursing may be considerable. While we found ways nurse leaders might better support their nurses, addressing stressors among leaders could benefit both leaders and nurses. The timely work of implementing workplace corrections to reduce nursing burnout and testing their impact is long overdue.

## Funding

This study was supported by American Medical Association.

## Conflicts of Interest

Dr. Mark Linzer is supported in this work by the American Medical Association (AMA), and for projects in burnout reduction, by IHI and Optum’s Office for Provider Advancement. These healthcare‐related projects are in the burnout field but represent minimal potential conflict for this study. Dr. Carolyn M. Porta is supported by the Pauline A Vincent Chair in Public Health Nursing; there should be no significant conflict from this role. Other authors have no conflicts of interest.

## Supporting Information

Supporting Table 1: *Header/title*: Representative questions from the Coping with Covid survey.

Supporting Table 2: *Header/title:* Participants (nurses, physicians, and other staff) in the current project on nurse burnout are represented in Supporting Figures 1A and 1B with respect to burnout and intent to leave; data are provided for participants for whom these data were available.

Supporting Figure 1A and 1B: *Header/title:* Comparisons by role (nurses, doctors, other clinical staff) of outcomes including burnout (Figure 1A) and intent to leave (Figure 1B) in the Coping with Covid study.

Supporting Figure 2: *Header/title:* Consort diagram for selecting 1315 nurse comments from those nurses without burnout.

Figure 2: Burnout item choices are as follows:

1 = I enjoy my work. I have no symptoms of burnout.

2 = I am under stress and don’t always have as much energy as I did, but I don’t feel burned out.

3 = I am beginning to burn out and have one or more symptoms of burnout, e.g., emotional exhaustion.

4 = The symptoms of burnout that I’m experiencing won’t go away. I think about work frustrations a lot.

5 = I feel completely burned out. I am at the point where I may need to seek help.

Supporting Figure 3: STROBE checklist for observational studies.

## Supporting information


**Supporting Information** Additional supporting information can be found online in the Supporting Information section.

## Data Availability

The data that support the findings of this study are available on reasonable request from the corresponding author. The data are not publicly available due to privacy or ethical restrictions.
