# Supplementary material for: Work Aspects Related to and Protective of Nurse Burnout During the Pandemic: A Cross‐Sectional Study
Source: J Nurs Manag. 2026 Feb 13;2026:1851095. doi: 10.1155/jonm/1851095 (PMC12905459; doi:10.1155/jonm/1851095)
Supplement: Supplementary file 1 — Supporting Information Additional supporting information can be found online in the Supporting Information section. [file JONM-2026-1851095-s001.zip › Supplementary Figure 1A and 1B Title.docx]

Supplementary Figure 1A and 1B. Comparisons by role (nurses, doctors, other clinical staff) of outcomes including burnout (Figure 1A) and intent to leave (Figure 1B) in the Coping with Covid study.
