# Supplementary material for: Work Aspects Related to and Protective of Nurse Burnout During the Pandemic: A Cross‐Sectional Study
Source: J Nurs Manag. 2026 Feb 13;2026:1851095. doi: 10.1155/jonm/1851095 (PMC12905459; doi:10.1155/jonm/1851095)
Supplement: Supplementary file 1 — Supporting Information Additional supporting information can be found online in the Supporting Information section. [file JONM-2026-1851095-s001.zip › Supplementary Figure 2 Title and Legend.docx]

Supplementary Figure 2.

Supplementary Figure 2 header/title: Consort diagram for selecting 1315 nurse comments from those nurses without burnout.

Supplementary Figure legend:

Burnout item choices (choices 1 or 2 = no burnout):

1=I enjoy my work. I have no symptoms of burnout.

2= I am under stress, and don't always have as much energy as I did, but I don't feel burned out.

3= I am beginning to burn out and have one or more symptoms of burnout, e.g. emotional exhaustion.

4=The symptoms of burnout that I'm experiencing won't go away. I think about work frustrations a lot.

5=I feel completely burned out. I am at the point where I may need to seek help.
