# Supplementary material for: Work Aspects Related to and Protective of Nurse Burnout During the Pandemic: A Cross‐Sectional Study
Source: J Nurs Manag. 2026 Feb 13;2026:1851095. doi: 10.1155/jonm/1851095 (PMC12905459; doi:10.1155/jonm/1851095)
Supplement: Supplementary file 1 — Supporting Information Additional supporting information can be found online in the Supporting Information section. [file JONM-2026-1851095-s001.zip › Supplementary Table 1.docx]

Supplementary Table 1: Representative questions from Coping with Covid survey.

Coping with Covid-19 for Caregivers Survey Items used in current study:

**Stress and Burnout:**

*Stress*: The stress I experienced today was minimal, modest, high, or very high

*Burnout**: Using your own definition of “burnout,” please choose one of the answers below:

1. I have no symptoms of burnout.
2. I am under stress, but I don’t feel burned out.
3. I am beginning to burn out and have one or more symptoms of burnout, e.g. emotional exhaustion.
4. The symptoms of burnout that I’m experiencing won’t go away. I am very burned out now.
5. I feel completely burned out. I am at a point where I may need to seek help.

**Potential burnout predictors and mitigators:**

*Fear*: I worry about exposing myself and my family to COVID (not at all, somewhat, moderately, to a great extent)

Due to the impact of COVID 19 I am experiencing the following:

*Anxiety or depression*: (not at all, somewhat, moderately, to a great extent)
*Work overload*: (not at all, somewhat, moderately, to a great extent)

*Concerns about childcare* (not at all, somewhat, moderately, to a great extent)

*Meaning and purpose*: Being a part of the COVID-19 response has increased my sense of meaning and purpose (not at all, somewhat, moderately, or to a great extent)

*Feeling valued***: I feel valued by my organization (not at all, somewhat, moderately, to a great extent)

**Work intentions*****:

*Intention to reduce hours*: What is the likelihood that you will reduce the number of hours you devote to

clinical care over the next 12 months? (None, slight, moderate, likely, definitely)

*Intention to leave*: What is the likelihood that you would leave your practice within two years? (None, slight, moderate, likely, definitely)

**Open ended comment:**
*What else would you like to tell us* about your experience during the COVID 19 crisis?

*Burnout question added April 30, 2020. **Feeling valued question added Fall of 2020. ***Work intention questions added summer, 2020.
