# Supplementary material for: Work Aspects Related to and Protective of Nurse Burnout During the Pandemic: A Cross‐Sectional Study
Source: J Nurs Manag. 2026 Feb 13;2026:1851095. doi: 10.1155/jonm/1851095 (PMC12905459; doi:10.1155/jonm/1851095)
Supplement: Supplementary file 1 — Supporting Information Additional supporting information can be found online in the Supporting Information section. [file JONM-2026-1851095-s001.zip › Supplementary Table 2.docx]

Supplementary Table 2: Participants (nurses, physicians and other staff) in current project on nurse burnout represented in Supplementary Figures 1A and 1B with respect to burnout and intent to leave; data provided for participants for whom these data were available.
