# Supplementary figures and images for: Work Aspects Related to and Protective of Nurse Burnout During the Pandemic: A Cross‐Sectional Study
Source: J Nurs Manag. 2026 Feb 13;2026:1851095. doi: 10.1155/jonm/1851095 (PMC12905459; doi:10.1155/jonm/1851095)

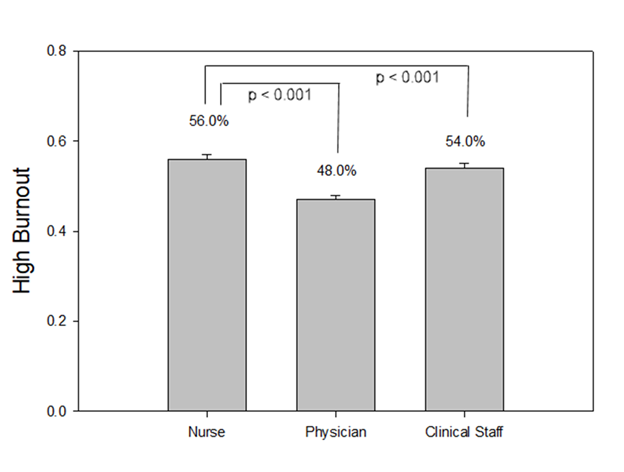

Supplement: Supplementary file 1 — Supporting Information Additional supporting information can be found online in the Supporting Information section. [file JONM-2026-1851095-s001.zip › Supplementary Figure 1A.png]

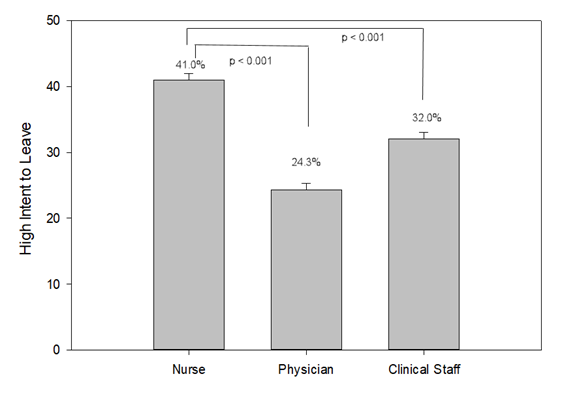

Supplement: Supplementary file 1 — Supporting Information Additional supporting information can be found online in the Supporting Information section. [file JONM-2026-1851095-s001.zip › Supplementary Figure 1B.png]

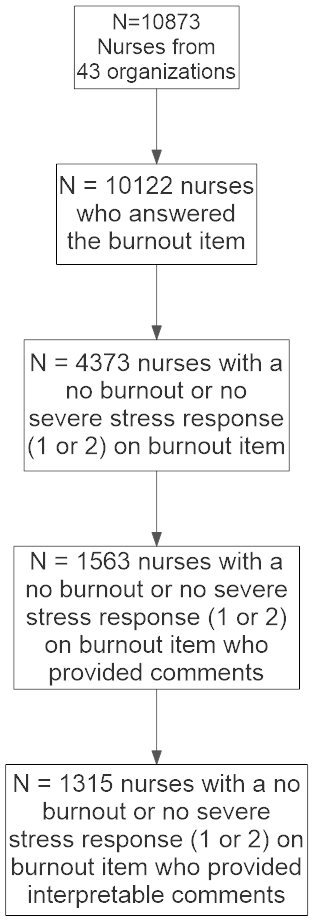

Supplement: Supplementary file 1 — Supporting Information Additional supporting information can be found online in the Supporting Information section. [file JONM-2026-1851095-s001.zip › Supplementary Figure 2.png]
